# Benefits of opportunistic screening for sexually transmitted infections in primary care

**DOI:** 10.1503/cmaj.210604

**Published:** 2021-04-19

**Authors:** Troy Grennan, Darrell H.S. Tan

**Affiliations:** British Columbia Centre for Disease Control (Grennan); Department of Medicine, Division of Infectious Diseases, University of British Columbia (Grennan), Vancouver, BC; Division of Infectious Diseases (Tan), St. Michael’s Hospital; Department of Medicine (Tan), University of Toronto, Toronto, Ont.

**KEY POINTS**
- A paucity of evidence means that guidance on how to approach testing for common sexually transmitted infections (STIs) in primary care is sparse.
- The recommendation of a new guideline issued by the Canadian Task Force on Preventive Health Care — to screen, once yearly, sexually active people younger than 30 years for chlamydia and gonorrhea — is justified and welcome, and its benefits will extend beyond case-finding.
- Given that STIs are often asymptomatic, may lead to serious sequelae, generate substantial onward transmission, and can be an independent risk factor for HIV acquisition, the increased diagnostic yield resulting from opportunistic screening may be invaluable.
- Regular opportunistic screening also has the potential to normalize conversations about sexual health, sexual orientation and STIs between clinicians and patients and thereby reduce stigma.

An epidemic of the bacterial sexually transmitted infections (STIs) syphilis, chlamydia and gonorrhea is taking hold worldwide, with the World Health Organization estimating that nearly 1 million people are infected daily with a curable STI.^1^ Canada has seen increases of more than 160% over the last decade.^2^ A critical component of STI control is testing, but a paucity of evidence on how best to do this, particularly with respect to screening frequency, means recommendations on the topic are scarce.

In a related guideline issued by the Canadian Task Force on Preventive Health Care, Moore and colleagues provide a recommendation for screening for chlamydia and gonorrhea in primary care.^3^ This publication updates national guidance on screening from the Public Health Agency of Canada,^4^,^5^ as well as guidelines by the Canadian Task Force on the Periodic Health Examination,^6^ which were last updated in 1996. The authors outline their rigorous use of the Grading of Recommendations Assessment, Development and Evaluation (GRADE)^7^ system, a widely accepted methodology for the development of clinical practice guidelines, including a systematic review of the literature, a 2-phase patient engagement process, and a transparent discussion of the benefits, harms and implementation considerations.

Given the paucity of available data, the guideline provides only 1 conditional recommendation, based on very low-certainty evidence: primary care providers should, once yearly, opportunistically screen sexually active individuals younger than 30 years who are not known to be part of a high-risk group for chlamydia and gonorrhea. Its authors argue this screening may confer an “uncertain but potentially important” benefit, such as the prevention of pelvic inflammatory disease in females.

One strength of the recommendation is that the authors intentionally extend their screening recommendation to individuals up to age 29 years (compared with the previous cut-off of 25 yr), to ensure that those with the highest STI rates are captured. This increase is justified by data showing recent increases in rates of STIs among people aged 25–29 years. For example, since 2012, the rate of gonorrhea in Canada in this age range has consistently been higher than among people aged 15–19 years, with data from 2017 showing 264 cases per 100 000 population versus 151 per 100 000, respectively.^2^

Another strength is the authors’ emphasis on an opportunistic approach to screening. Although the randomized controlled trials available did not explicitly examine this approach, such a strategy is logical because it capitalizes on existing health care interactions to seek health benefits that patients value. Experience from the HIV epidemic reinforces the value of an opportunistic approach; too often, our health system identifies people living with HIV for the first time at late stages of the disease, only to find that they had had multiple “missed opportunities” for diagnosis during earlier, unrelated health care encounters.^8^,^9^ Given that STIs are often asymptomatic, may lead to serious sequelae (e.g., pelvic inflammatory disease, infertility, disseminated gonococcal infection), generate substantial onward transmission, and can be an independent risk factor for HIV acquisition,^10^,^11^ the increased diagnostic yield resulting from such screening may be invaluable.

A final potential benefit of the authors’ recommendation is its potential to normalize conversations about sexual health and STIs between clinicians and patients, which have long been marred by stigma and shame. Offering screening may help patients feel that they “have permission” to discuss health issues that may seem difficult to talk about. The guideline authors’ literature review identified stigmatization and anxiety about STI-related outcomes as one of the few adverse effects of STI screening. Yet, by intentionally asking about STI screening during unrelated health care encounters, clinicians can send powerful signals to their patients that may counteract stigma.^12^

Opportunistic STI screening in primary care settings represents an important step in the right direction. However, a short-coming is that the guideline recommendation specifically excludes groups at higher risk for STI acquisition, begging the question: How will providers ever find those higher-risk individuals? For instance, several studies have shown that up to 90% of gay, bisexual and other men who have sex with men — a group disproportionately affected by STIs — often do not disclose their sexual orientation to their providers.^13^ Recognizing the stigma associated with sexual health, providers should not only offer opportunistic STI screening to their lower-risk patients, but precede this offer with nonjudgmental efforts to ascertain the patient’s true risk profile. In the absence of such a conversation, other aspects of sexual health for individuals at higher risk — such as HIV testing or the provision of extragenital (i.e., rectal or pharyngeal) testing for STIs — may be easily overlooked.

Bacterial STIs remain a substantial global public health concern, yet basic questions — including who, when and how often to screen — remain unanswered. Indeed, the most concerning issue emerging from the work that informed the new guideline is the paucity of good-quality research on chlamydia and gonorrhea screening. Despite a comprehensive and methodologically rigorous approach to their systematic review, the authors were still left with few good-quality studies, many of which were not particularly applicable to real-world primary care settings (e.g., the use of mailed invitations to screening). The guideline developers should be lauded for undertaking such important work in an underinvestigated area, but their findings should also be a call to action for clinicians, researchers and public health professionals to make space for conversations about sexual health and STIs, and reprioritize these issues on their research agendas.
